# Bariatric surgeons' experiences of working in the first year of the pandemic

**DOI:** 10.1002/osp4.655

**Published:** 2023-01-06

**Authors:** Yitka N. H. Graham, Kamal Mahawar, Rishi Singhal, Brij Madhok, Wah Yang, Manel Riera, Pedro Martinez‐Duartez, Sjaak Pouwels, Mitesh Sharma, Catherine Hayes

**Affiliations:** ^1^ Faculty of Health Sciences and Wellbeing School of Nursing and Health Sciences Helen McArdle Nursing and Care Research Institute University of Sunderland Sunderland UK; ^2^ Bariatric Surgical Unit South Tyneside and Sunderland NHS Foundation Trust Sunderland UK; ^3^ Faculty of Psychology University of Anahuac Mexico Naucalpan Mexico; ^4^ Upper GI Unit University Hospital Birmingham NHS Foundation Trust Birmingham UK; ^5^ East Midlands Bariatric and Metabolic Institute University Hospitals of Derby and Burton NHS Foundation Trust Derby UK; ^6^ Department of Metabolic and Bariatric Surgery The First Affiliated Hospital of Jinan University Guangzhou China; ^7^ Bariatric Surgical Unit The Shrewsbury and Telford Hospitals NHS Trust Shrewsbury UK; ^8^ Austral University Hospital Buenos Aires Argentina; ^9^ Department of Intensive Care Medicine Elisabeth Tweesteden Hospital Tilburg The Netherlands; ^10^ Department of Surgery Agaplesion Bethanien Krankenhaus Frankfurt Am Main Hessen Germany

**Keywords:** bariatric surgery, covid‐19, mental health, pandemic, wellbeing

## Abstract

**Background:**

The first year of the Covid‐19 pandemic saw drastic changes to bariatric surgical practice, including postponement of procedures, altered patient care and impacting on the role of bariatric surgeons. The consequences of this both personally and professionally amongst bariatric surgeons has not as yet been explored.

**Aims:**

The aim of this research was to understand bariatric surgeons' perspectives of working during the first year of the pandemic to explore the self‐reported personal and professional impact.

**Methods:**

Using a retrospective, two phased, study design with global participants recruited from closed, bariatric surgical units. The first phase used a qualitative thematic analytic framework to identify salient areas of importance to surgeons. Themes informed the construction of an on‐line, confidential survey to test the potential generalizability of the interview findings with a larger representative population from the global bariatric surgical community.

**Findings:**

Findings of the study revealed that the first year of the pandemic had a detrimental effect on bariatric surgeons both personally and professionally globally.

**Conclusion:**

This study has identified the need to build resilience of bariatric surgeons so that the practice of self‐care and the encouragement of help‐seeking behaviors can potentially be normalized, which will in turn increase levels of mental health and wellbeing.

## 1 INTRODUCTION

Following the World Health Organization's declaration of coronavirus as a global epidemic in March 2020,^1^ the International Federation for the Surgery of Obesity and Metabolic Disorders (IFSO) released guidelines recommending the postponement of bariatric surgery in order to protect both patients and healthcare professionals working in this clinical discipline.^2^ As a direct result of the elevation to pandemic status, countries were subject to lockdowns including social distancing measures designed to control the spread of infection. For bariatric surgical teams, everyday practice was, disrupted, significantly impacting on the provision of bariatric surgery and patient care^3^ and leading to unprecedented challenges. Some bariatric surgeons, like many other healthcare professionals, were redeployed to other areas of clinical care, furloughed, or left without a source of income.^4^ Many countries moved with immediacy from face‐to‐face patient consultations to video and telephone consultations,^5^ which occurred virtually overnight, which meant that bariatric surgeons had only minimal time to adjust to new ways of working. The ongoing and complex nature of working in an environment dominated by COVID‐19 was a source of stress, especially for bariatric surgeons, which could potentially result in reduced mental wellbeing.^6^, ^7^

The psychological engagement of staff within the workplace is a fundamental issue for all healthcare providers. Presenteeism is defined as people attending work despite not being mentally engaged, which can be attributed to both physical or mental ill health^8^ One of the causative factors of presenteeism is the impact of often unexpected and enforced levels of psychosocial stress, characterized by workplace change and uncertainty^9^ such as with the Covid‐19 pandemic.^10^ It is these aspects of psychosocial stress that mean continuing to work in a world dominated by Covid‐19. These may have consequences for optimal surgical performance in relation to the potential for emotional labor, moral injury and compassion fatigue,^4^ all of which may lead to burnout levels of which are evidenced to be high among all surgical specialties.^11^ The impact of Covid‐19 on bariatric surgical practice has served to highlight the necessity of understanding the mental wellbeing of bariatric surgeons if they are to be optimally supported in their roles. For many reasons, including the social construct of a surgeon as one who is in control and invokes trust,^12^ mental health issues are not always easy to discuss, there are acknowledged low presentation rates of poor mental wellbeing among surgeons.^13^ The aim of this study was to explore bariatric surgeons' perspectives of working during the first year of the pandemic to understand the impact this has had on surgeons both personally and professionally. This may inform planning and provision of long‐term support and care for the wellbeing of bariatric surgeons.

## 2 METHODS

This study used a retrospective, two phased, study design with participants recruited from closed, global bariatric surgical professional Facebook® groups (The Upper Gastrointestinal Surgery Society [TUGSS]). Eligibility criteria for inclusion in the study were practicing bariatric surgeons. Other members of the bariatric surgical multidisciplinary team, retired surgeons or trainees were excluded from this study to be able to focus on surgeons and their wellbeing.

The first phase used a qualitative thematic analytic framework, to identify salient areas of importance to the participants. Qualitative research is appropriate for understanding the why's and how's of social phenomena, and especially useful when exploring areas where little is known on the subject.^14^, ^15^ With the long‐term impact of COVID‐19 on the wellbeing of bariatric surgeons a relatively unknown entity, this methodological approach was deemed to be appropriate to conduct the first phase of the study. A thematic analytic framework was used to analyze the interview data^15^ guided by a framework adaptation of the method advocated by Braun and Clarke.^16^

Data were collected through confidential, face to face semi‐structured interviews, assisted by a topic guide. The interviews took place video (Teams® or Zoom®) at a time convenient to each participant. Each participant provided written consent; the interview was audio‐recorded and transcribed verbatim and lasted between 20–45 min. The interviews were all carried out by the same female researcher, who was experienced in qualitative research and works as a researcher in a bariatric surgical unit.

A constant comparative approach was used with the interview data, meaning data collection and analysis were undertaken concurrently, with analysis guiding further sampling^10^. The researcher also took written notes during the interview, to document any areas of interest, and identify areas of importance to the participants, which could be to be explored further during the interview, clarifying any ambiguity to ensure that interpretation of data was veracious to the participants' experience and to minimize potential for researcher bias.

Data collection and analysis continued until no new concepts were identified, meaning data was saturated and recruitment to phase 1 could be ceased. The concepts were discussed with the research team and a consensus on the core set of emergent themes was identified and agreed between all researchers. The findings of the thematic analysis were used to construct the survey for the second phase, which formed the questions for the on‐line, confidential survey which would test the generalizability of the interview findings with a larger cohort of bariatric surgeons. Consent was implied for the survey; completing the survey was taken as consent to participate in the study.

Data were collected from May–December 2021. Ethical approval was granted from the University of Sunderland Research Ethics Committee.

## 3 RESULTS

A total of 120 surgeons took part in the study, with five recruited to phase one (interviews) and 115 respondents to the second phase survey. For the interviews, participants (2 females, 3 male) consented to be interviewed. Participants were from Italy, China, Spain, France, and Chile and worked in public hospitals, with two engaging in private practice. Participants had been practicing bariatric surgeons for between 6 and 14 years.

There were five themes constructed from the interview data (See Table 1), which informed the construction of questions for the survey, which was the second phase of the study.

**TABLE 1:** Five themes constructed from interviews

Theme	In vivo quotes
Acknowledging the uncertainties of the pandemic personally and professionally	*At first I felt really bad and really sad because we couldn't help all our patients, the patients living with obesity, and that was very dangerous for them. For example, oncology patients, the first time the oncology surgeries didn't stop, I mean I think that everybody thinks that oncology are more important, of course they're important but bariatric and patients living with obesity are very important, they have a lot of comorbidities which are risks to their health, so bariatric surgery is so important, as important as a cancer, in my opinion. That was really, really sad* (Participant E)
Negotiating a new normal for surgical practice in response to the pandemic	*I think it's very important you know the point of view about the society, hospitals and health system, about obesity, stigma and the role of bariatric surgery as a medical treatment for weight‐loss and comorbidity improvement. Many professionals don't think that obesity is, very important. It is another pandemic and we have to make a lot of effort to change how society thinks about obesity as a pandemic. Obesity is dangerous, more than cancer in my opinion. Around the world, there are many more deaths related to obesity and the comorbidities of obesity. The‐word cancer makes a lot afraid, all the population is very, very conscious about cancer but not about obesity. We have to work very, very hard to change opinions and increase the number of bariatric surgeries that we perform globally* (Participant C)
Balancing home and work boundaries	*You're afraid for your family every time you get home when you're attending patients with COVID. You get home, you kiss your wife, you kiss your children, and you say, “My God, I hope not to be transmitting the disease to them.” Hopefully that didn't happen but I don't know if it's going to happen yet. I am vaccinated, I already had the disease, but I don't know if I'll transport the disease with me. You have to leave your work problems at work but it was very difficult* (Participant B)
Being aware of the need for personal wellbeing	*We had some really bad cases due to COVID. I remember a pregnant woman who had a caesarean section, she was in our department and I was on duty for general surgery. At the end she died because she had post‐operative bleeding you know, with COVID problems at the end. The whole organization was too late, so it wasn't my patient, but being the general surgeon on duty this day, she was COVID positive in our department, things like this obviously hurt. You see how important life is, you remember again how important life is and that it can finish immediately. And you have to reflect on the impact of things like this on your wellbeing, and try to deal with this as best you can* (Participant C)
Reflecting on potential long‐term impact of the pandemic on surgeons and surgical practice	*Many surgeons are saying that a lot of times people feel that surgeons, they have this impression, are very strong, you have to trust them and they have a face they present to people of what everybody thinks a surgeon should look like. Behind the face there is a heart, there is a soul, there is a person in pain because of everything they've seen. It's like stress that you haven't had before in your life but either you are not doing surgery and you have to do something else, and that is very stressful because a bariatric surgeon wants to do bariatric surgery and when they can't do it that's stressful and they know the patients are suffering. This can affect our health in the future* (Participant E)

There were 115 respondents to the on‐line survey in Phase Two, representing 28 countries (see Table 2). Most participants were from the United Kingdom (13.9%, *n* = 16) India (9.5%, *n* = 11), China (8.7%, *n* = 9) and Turkey (8.7%, *n* = 9).

**TABLE 2:** Self‐reported years in bariatric surgical practice

Years working in bariatric surgical practice	Respondents (*n* = 114)
<Than 3 years	11.4% (*n* = 13)
3–5 years	17.5% (*n* = 20)
6–8 years	14.9% (*n* = 17)
9–11 years	13.1% (*n* = 15)
12–14 years	12.2% (*n* = 14)
15–17 years	12.2% (*n* = 14)
18–20 years	6.1% (*n* = 7)
>20 years	12.2% (*n* = 14)

The majority of the respondents were male (87.9%, *n* = 102) and 11.2% (*n* = 13) stated their gender as female. One fifth of the respondents had been practicing as a bariatric surgeon for 3–5 years (17.5%, *n* = 20) and there was consistent representation across years in practice, with only 6.1% (*n* = 7) in practice between 18 and 20 years (see Table 2).

The modal age range of participants was 40–44 years (33%, *n* = 26).

Participants reported their hospitals or clinics were public or state funded (62%, *n* = 72), private (54.3, *n* = 63), insurance‐led (10.3%, *n* = 12) or other (3.4%, *n* = 4) in accordance with their representative national healthcare systems.

Most respondents (88%, *n* = 103) reported that their country went into a national lockdown, with 7% (*n* = 9) stating no, and 3% (*n* = 4) reporting other. When asked if, and for how long their country went into lockdown in March 2020 for, over a third (39%, *n* = 46) stating 3–4 months 30% (*n* = 35) stated 1–2 months, 17% (*n* = 20) stated 5–6 months (17%, *n* = 20) and 12% (*n* = 15) stating other.

Following IFSO guidance to postpone bariatric surgery, respondents said their respective hospitals or clinics stopped bariatric surgery (43%, *n* = 50), all elective bariatric surgery ceased (23%, *n* = 27), bariatric surgery continued, but at a reduced level (16%, *n* = 19), bariatric surgery continued as normal (2%, *n* = 3) and 5% (*n* = 6) stating other.

To delineate the person from the profession, participants were asked how they felt on a personal level when Covid 19 was announced as a pandemic. The responses were free text, with 111 responses (3 declined to respond). The most common feeling (74%, *n* = 82) was that of worry, in the context of self, co‐workers and family. Nearly 14%% (*n* = 15) respondents felt that the situation would be short‐term for example, 2–3 months, stating they were not too worried. The remaining 12% (*n* = 13) reported feeling comfortable about the pandemic.

Participants were asked the same question, but in their professional capacity as a bariatric surgeon. Of the 113 responses (2 skipped the question), over three quarters of respondents (81%, *n* = 91) felt worried about the impact the pandemic, with the main areas of concern being patient care, effect on performance of bariatric surgical procedures and risk of infection. Fifteen percent (*n* = 13) felt that they had to adapt to the situation, and felt comfortable doing so, but acknowledged an element of adaptation of working practice and environment. The remaining nine responses did not offer sufficient information to be included.

During the pandemic, some participants reported being transferred from bariatric surgery into other areas of care (see Table 3). The length of time spent in these positions varied and was not consistently reported.

**TABLE 3:** Changes that happened with daily work routine during the pandemic

Changes	Responses
I Did not see patients face to face	37% (*n* = 43)
I Consulted with patients by telephone	58% (*n* = 50)
I Consulted with patients by video (Zoom®, Skype® etc.)	48% (*n* = 41)
I Worked from home all the time	9% (*n* = 8)
I Worked from home part of the time	32% (*n* = 28)
I Worked in the hospital/clinic as normal, in bariatric surgery	31% (*n* = 27)
I Was transferred to another area in the hospital	18% (*n* = 15)
I Was furloughed (paid, but had to stay at home and not work)	3% (*n* = 3)
Other (please specify)	17% (*n* = 15)

The self‐reported main changes to pre‐ and post‐bariatric surgical care were stated as follows. Pre‐surgically, 62% (*n* = 72) said patients remained on waiting lists, but with no date for surgery, 59% (*n* = 68) stated patients received telephone support, and 46% (*n* = 53) received video support. Nearly half (41%, *n* = 48) said that patients were advised to continue with pre‐operative advice. Face to face patient support meetings ceased (34%, *n* = 40), with 23% (*n* = 27) running patient support groups by video. Patients were taken off the waiting list by 5% (*n* = 6) of respondents, and 4% (*n* = 5) reported other.

Post‐surgically, changes included an increase in telephone follow up (72% *n* = 84) 46% (*n* = 54) used video follow‐up, 43% (*n* = 51) stopped all face‐to‐face appointments and 13% (*n* = 16) reported other.

The issues reported to cause participants to feel stressed or worried were reported as the wellbeing of their families (75%, *n* = 87), being unable to perform bariatric surgery (49%, *n* = 57), contracting Covid‐19 (46%, *n* = 54), wellbeing of post‐operative patients (37%, *n* = 44), wellbeing of colleagues (31%, *n* = 37), wellbeing of pre‐operative patients (30%, *n* = 35), finances (29%, *n* = 34), job security (27%, *n* = 32) and 5% (*n* = 6) reporting other.

The respondents were asked about their feelings about the personal impact of the pandemic across five areas of mental wellbeing, physical health, work/life balance, personal and professional relationships (see Table 4).

**TABLE 4:** Self‐reported personal impact of the pandemic

Personal impact	Responses (participants could select more than one area)
My mental wellbeing was not good	27.8% (*n* = 32)
My mental health was fine	51.3% (*n* = 59)
My physical health suffered	20% (*n* = 23)
My physical health was not affected	52.1% (*n* = 60)
My work/life balance suffered as a result of the pandemic	45.2% (*n* = 52)
My work/life balance did not suffer	28.6% (*n* = 33)
My personal relationships were negatively affected by the pandemic	26.9% (*n* = 31)
My personal relationships were not affected	41.7% (*n* = 48)
My relationships with colleagues became difficult	14.7% (*n* = 17)
My relationships with colleagues remained the same	48.6% (*n* = 56)
My relationships with colleagues has improved	13.9% (*n* = 16)
Other (please specify) The pandemic gave me a fresh perspective as there was a lot of time to think and re‐evaluate. It helped me to refocus on what I really wanted from my life. Unable to go to the gym affected weight gain My relationships with selective colleagues became more difficult There has been a lot of stress, but have maintained my sanity My financial security was impacted	4.3% (*n* = 5)

Participants were asked if enough is being done to support the overall wellbeing of bariatric surgeons, with 29% (*n* = 34) reporting there is support at work if they require this. Equally, 24% (*n* = 28) stated not having support, with the same number stating that awareness of mental wellbeing and resilience needs to be increased. Nearly one quarter (21%, *n* = 24) felt that personal mental wellbeing is a subject that bariatric surgeons tend not to speak about.

When asked if they felt their mental wellbeing has suffered because of the pandemic, 51% (*n* = 59) said no, and 48% (*n* = 55) stated yes. When asked if they had accessed any support for their wellbeing, formally or informally in the first year of the pandemic, 76% (*n* = 89) had not, but nearly one quarter (23%, *n* = 27) had.

At the time of data collection, 50% (=58) stated that bariatric surgical services were running, but not at pre‐pandemic levels, 46% (*n* = 54) reported that all bariatric surgical services were operational at pre‐pandemic levels, 4% (*n* = 5) stated services were not running and 1 reported “other”.

When asked what the future provision of bariatric surgery will look like, nearly half (46%, *n* = 52) stated that telephone and video consultations will remain, with less face to face appointments, and comparatively (24%, *n* = 28) it was felt that both services will return to how they were pre‐pandemic, or there will be an increase in bariatric surgical procedures performed. The remaining 5% (*n* = 6) stated they felt that less bariatric surgery will be performed.

In terms of their career as a bariatric surgeon as a result of the pandemic, 92% (*n* = 105) would continue in their role, with 3% (*n* = 4) considering a change to another type of surgical career, and 3% (*n* = 2) were considering leaving the profession and doing something else. Of the remaining two participants, one stated they would retire in the next year, and the other participant reported considering retiring earlier than planned.

Finally, participants were asked to comment on any aspect of the impact of the pandemic on bariatric surgery and anything that they felt would support bariatric surgeons as COVID‐19 continues to dominate patient care and provision of bariatric surgery. There were 35 responses to this question. Over half the respondents (20, *n* = 57%) stated bariatric surgeons needed support, both psychologically and pragmatically in terms of equipment, insurance, financial implications, increased recognition of bariatric surgery as a priority procedure, provision of training in both surgical and management of COVID‐19 and the need to work more collaboratively as a clinical specialty. Four respondents (*n* = 11%) commented on the place of other treatment or surgical options such as anti‐obesity drugs and robotic surgery going forward. Patient care issues such as lifestyle support, dealing with patients on waiting lists and highlighting the risks of obesity in a COVID‐19 context were reported by the remaining 10 respondents (3.5%).

## 4 DISCUSSION

Since this was the first global survey to examine and explore the impact of the pandemic on the practice of bariatric surgery and the mental wellbeing of bariatric surgeons 1 year into the pandemic it was necessary to revisit the results of the study in the light of the existing literature in similar and parallel fields, which were reflective of our own findings, despite not exploring exactly the same aspects of it. Over 2 years on, the burden of Covid‐19 on healthcare systems and health care workers has been substantial, and the ongoing complex nature of the pandemic's trajectory remains challenging. The long‐term impact and consequences are still yet largely unknown and unquantified.

The isolation and quarantine measures varied from country to country in terms of severity and length, and this may have influenced the reported variance in the provision of bariatric surgery during the first lockdown in March 2020.

Our findings consolidate the recognition of the importance of understanding the mental wellbeing of healthcare professionals in the pandemic, which has also been explored in studies on healthcare workers, surgical and clinical disciplines.^17^, ^18^ As many bariatric surgeons were redeployed or worked in other areas of care during the first year of the pandemic, it is important to examine the mental wellbeing of personnel from other areas of clinical care since collective psychological resilience is as important as individual psychological wellbeing in the context of patient care.

From a more generalist perspective than ours, a scoping review of the impact of the pandemic on surgical practice highlighted 66 studies which detailed the effects that Covid‐19 has had on all aspects of surgery, including precautions, complications, procedures, staffing and scheduling, and suggested that psychological support and training platforms were necessary for surgical teams.^19^ The situational and context specificities of bariatric surgical intervention meant several parallels could be made with our own findings in relation to this paper, alongside the meta‐analysis of the 66 studies outlined within it.

In terms of the address of specific mental health issues, a systematic review of 13 studies with a total of 33,062 healthcare workers as participants found anxiety (pooled prevalence of 23.2% in 12 studies), depression (pooled prevalence of 22.8% in 10 studies) and insomnia (pooled prevalence of 38.9% across five studies), with a subgroup analysis revealing higher rates of affective symptoms in female healthcare professionals and nursing, suggesting potential gender and professional differences which warrant further investigation.^20^ This bears a striking similarity to the findings of our own study which revealed correspondingly high levels of despondency and a reported diminished sense of personal and collective resilience. A further systematic review of 76 studies on the mental wellbeing of healthcare workers in pandemics found risk factors for poor mental health included working in front line healthcare situations, female, being a nurse, lack of adequate personal protective equipment, longer shifts, lack of knowledge of the virus, inadequate training, less years of experience in healthcare than their clinical counterparts, lack of social support, and a history of quarantine.^21^ What this study highlighted was the need to further examine the experiences of bariatric surgical teams to establish whether the signature discipline of nursing practice provided correspondingly reported issues for surgery, or whether the challenges of being a bariatric surgeon were in some way specific to the profession.

In terms of the ecological validity of our own study, and its potential transferability to other contexts, a survey of 220 Latin‐American trauma surgeons reported that 127 (57.7%) participants felt emotionally overextended, anxious and exhausted, especially due to the COVID‐19 social isolation and uncertainties of how long it will take for life to “return to normal”.^22^ The universal experience of this phenomenon was tangible in the outcomes of our own study and it is notable too, that a review of the impact of Covid‐19 on vascular surgery reported high levels of anxiety, attributed to exposure to the virus, moral injury, changes to practice and financial issues,^23^ all of which were noted in our study.

Ear, Nose and Throat (ENT) specialists are a high‐risk group for Covid‐19 and potentially increased psychological harm. An Irish survey of 38 ENT specialists found that 34% (*n* = 13) screen positive for anxiety, and 84% (*n* = 32) felt that they had increased exposure to the virus compared with other specialties, with 32% (=12) feeling unable to protect themselves from Covid‐19.^24^ The rates of burnout and increased risk of poor outcomes for 684 orthopedic surgeons in a cross sectional survey across five countries was found to be high irrespective of healthcare system.^25^ This has important repercussions for bariatric surgeons who may experience parallel issues in terms of the context specificity of their own work.

The pandemic has provided a context of complex ambiguity for the discipline of bariatric surgery, which has entailed adaptation from usual practice and then a gradual re‐adaptation back to a “new normal” which has ensured no direct resolution of psychological challenge. In terms of emotional labor, for many, this has created a culture of uncertainty, where each iterative wave of the virus presents new challenges and the need for constant change. In terms of the psychological burden this places upon all bariatric surgeons globally, this is clearly an issue for address if a sustainable number of colleagues are to remain in active surgical roles.

Mental health and wellbeing is an area of health which is often difficult for healthcare professionals to admit and/or discuss^26^ and may result in internalization of low mood and thoughts^27^ and may be a barrier to help‐seeking. Results from our study also reflected issues which may have direct relevance to the psychological wellbeing of surgeons in relation to their capacity for emotional labor within the context of bariatric care. This raises important implications for health promotion and education within the global bariatric surgical settings and in relation to the monitoring and future sustainability of the bariatric surgical workforce.

## 5 CONCLUSION

This is the first study to examine the impact of the first year of Covid 19 on bariatric surgeons and surgical practice. The findings are limited to those surgeons who are members of TUGS, and of the countries they work in. All data for the surveys was self‐reported and anonymous which means that further checks of content validity were not part of the study. There was a gender imbalance in our study, with only 11% (*n* = 13) of women participating in the study, so the collected data is potentially biased toward experiences of male surgeons. Given the reported high rates of poor wellbeing in females in other healthcare disciplines, the experiences of female bariatric surgeons should be explored in further research to ascertain whether gender disparities are impacting upon female surgeons to the same extent or whether they differ.

As yet, the long‐term consequences of the pandemic on the mental wellbeing of bariatric surgeons is not known. This study captures the experiences of bariatric surgeons at a crucial time point, which may assist to provide a greater understanding of the context of the pandemic and its effect on the mental wellbeing of bariatric surgeons during an unprecedented and difficult time.

There needs to be continued acknowledgment of the need to build individual and collective resilience among bariatric surgeons so that the practice self‐care and the encouragement of help‐seeking behaviors can potentially be normalized in an effort to increase mental wellbeing for the long‐term sustainability of the global bariatric surgical workforce.

## AUTHOR CONTRIBUTIONS

Yitka N. H. Graham and Kamal Mahawar designed the study, all authors contributed to data collection and were involved in the writing of the paper and had final approval of the submitted and published versions.

## CONFLICT OF INTEREST

The authors have no conflicts of interest to declare.
